# Monitoring Turkish white cheese ripening by portable FT-IR spectroscopy

**DOI:** 10.3389/fnut.2023.1107491

**Published:** 2023-02-06

**Authors:** Hulya Yaman, Didem P. Aykas, Luis E. Rodriguez-Saona

**Affiliations:** ^1^Department of Food Science and Technology, The Ohio State University, Columbus, OH, United States; ^2^Department of Food Processing, Bolu Abant Izzet Baysal University, Bolu, Türkiye; ^3^Department of Food Engineering, Adnan Menderes University, Aydin, Türkiye

**Keywords:** cheese ripening, Turkish white cheese, FT-IR, extraction methods, organic acids, free amino acids, free fatty acids

## Abstract

The biochemical metabolism during cheese ripening plays an active role in producing amino acids, organic acids, and fatty acids. Our objective was to evaluate the unique fingerprint-like infrared spectra of the soluble fractions in different solvents (water-based, methanol, and ethanol) of Turkish white cheese for rapid monitoring of cheese composition during ripening. Turkish white cheese samples were produced in a pilot plant scale using a mesophilic culture (*Lactococcus lactis* subsp. lactis, *Lactococcus lactis* subsp. cremoris), ripened for 100 days and samples were collected at 20-day intervals for analysis. Three extraction solvents (water, methanol, and ethanol) were selected to obtain soluble cheese fractions. Reference methods included gas chromatography (amino acids and fatty acid profiles), and liquid chromatography (organic acids) were used to obtain the reference results. FT-IR spectra were correlated with chromatographic data using pattern recognition analysis to develop regression and classification predictive models. All models showed a good fit (R_Pre_ ≥ 0.91) for predicting the target compounds during cheese ripening. Individual free fatty acids were predicted better in ethanol extracts (0.99 ≥ R_Pre_ ≥ 0.93, 1.95 ≥ SEP ≥ 0.38), while organic acids (0.98 ≥ R_Pre_ ≥ 0.97, 10.51 ≥ SEP ≥ 0.57) and total free amino acids (R_Pre_ = 0.99, SEP = 0.0037) were predicted better by using water-based extracts. Moreover, cheese compounds extracted with methanol provided the best SIMCA classification results in discriminating the different stages of cheese ripening. By using a simple methanolic extraction and collecting spectra with a portable FT-IR device provided a fast, simple, and cost-effective technique to monitor the ripening of white cheese and predict the levels of key compounds that play an important role in the biochemical metabolism of Turkish white cheese.

## 1. Introduction

Turkish white cheese, Kashar cheese, and Tulum cheese are the most produced industrial cheeses among traditional cheese varieties in Turkey (1). These cheeses are either consumed fresh or ripened according to the traditional characteristics of the product. For example, fresh classic white cheese produced from cow’s milk is taken to the market with minimum a three-week ripening, while Ezine-type white cheese can have a ripening period of up to 6 months (2). Therefore, determining the quality characteristics of the cheese is important to monitor the degree of ripening of the cheese. The biochemical reactions and their importance for the industry have been explained in detail by many researchers (3–5). Cheese ripening involves various biochemical and microbiological changes by the metabolism of starter cultures, indigenous, clotting, adjunct enzymes, and ripening accelerating agents. The biochemical reactions that occur during cheese ripening can be classified into three main categories: (1) the catabolism of residual lactose and citrate into organic acids and other components (glycolysis); (2) the catabolism of proteins into amino acids and other amin products (proteolysis); and (3) the catabolism of the fat into fatty acids and other further lipolysis compounds (3, 4).

The degree of importance of these metabolites during the ripening period may vary according to the production method and type of cheese. While lipolysis plays a more active role in cheeses with high-fat content or ripened with mold and fatty acids formed during ripening (6–8), proteolysis is responsible for texture development, functionality, and flavor improvement through amino acids and small peptides formation (9, 10). The residual lactose is converted to organic acids and other further breakdown compounds by glycolysis depending on the used starter culture or indigenous microbiota (6, 11–14).

Monitoring the level of these molecules during ripening provides information about the development of ripening and product quality. Determination of these biochemical changes by analytic methods is time-consuming, laborious, expensive, and involves extensive chemical use also complex analysis methods with expensive equipment, especially chromatographic techniques (15). The disadvantages can be overcome using new, rapid, portable, and simple methods based on Fourier-transform infrared (FT-IR) spectroscopy. Previously, it was found a high correlation coefficient (*R* > 0.90) for the prediction of acetic, propionic, and butyric acid contents using the FT-IR spectra for Swiss cheese with a novel sample preparation method of water-soluble extractions (16). Similarly, the free amino acid content of cheddar cheese samples was determined from the water-soluble extract of cheese with a high correlation with FT-IR spectra (17, 18). Furthermore, the primary composition of Turkish white cheese through ripening were examined by three vibrational spectroscopy methods (NIR, FT-IR, and Raman), and the suitability of the analytic method has been demonstrated (19).

Our objective was to evaluate the unique fingerprint-like infrared spectra of target soluble compounds in Turkish white cheese, including amino acids (GC-MS), fatty acids (GC-FID), and organic acids (HPLC-PDA), to evaluate their influence on the metabolic profile throughout the ripening period. Solvents (water, methanol, and ethanol) with different polarities were used to extract target compounds from Turkish white cheese samples and these fractions were characterized using a metabolomics study for biomarkers to enable rapid monitoring of the cheese ripening process.

## 2. Materials and methods

### 2.1. Manufacture of Turkish white cheese

Whole cow milk (pH 6.67) was standardized (1:1 protein: fat ratios) and then pasteurized (at 65°C for 30 min) and dropped the temperature to 32°C for clotting. Mesophilic culture-specific for Turkish white cheese (*Lactococcus lactis* subsp. lactis, *Lactococcus lactis* subsp. cremoris) (Choosit MA11, Danisco, France) was added to milk with a ratio of 0.002% and fermented until the pH reached 6.4. In addition, 0.2% CaCl2 and rennet enzyme (CHY-MAX, Chr. Hansen, Hoersholm, Denmark) were added and rested for one and a half hours. Afterward, the clot formed was cut into 1 cm cubes, waited for 30 min for syneresis, and transferred into the cheesecloth. Subsequently, the pressure was applied as 20 kg weights for each 100 kg of cheese milk for four hours then teleme was obtained and it was cut into 7 × 7 cm cubes and waited for 12 h in 16% NaCl brine at 20°C. Cheese blocks were placed in 12% brine in an airtight Ziplock bag and stored at 4°C until further analysis. Cheese samples were ripened for 100 days and analyzed at each 20 days interval.

A batch of Turkish white cheese produced at The Ohio State University Dairy Plant was cut into 36 cubes and then divided into six groups to be used for analysis at each ripening time (1, 20, 40, 60, 80, and 100 days). The cheese production was replicated and as a total of 72 cheese blocks were manufactured in two repetitions.

### 2.2. Sample preparation for the FT-IR analysis

Approximately 20 g Turkish white cheese sample was blended with liquid nitrogen and ground cryogenically using a Waring Lextra 2 speed blender (East Windsor, NJ, USA) to produce a fine cheese powder.

Water-soluble extracts followed the procedure described by Subramanian et al. (18) and were prepared by mixing 0.1 g of the cheese powder with 0.5 mL of distilled water. The mixture was sonicated by an ultrasonic water bath (Fisher Scientific, Pittsburgh, PA) for 10 s to extract water-soluble components, and then 0.5 mL chloroform was added to remove the complex fats, vortexed for 30 s, and the mixture was centrifuged at 15,700 × *g* for 3 min at 25°C (Megafuge 8, Thermo Fisher Scientific, Waltham, MA, USA). Supernatants (200 μL) were mixed with 200 μL absolute ethanol to precipitate the complex proteins in the mixture and centrifuged at 15,700 × *g* for 3 min at 25°C. The supernatant was employed for further spectroscopic analysis.

Methanol and ethanol extracts were prepared by mixing 0.1 g of the cheese powder with either 1.0 mL of 100% methanol or 100% ethanol solutions. The mixtures were sonicated by using an ultrasonic water bath (Fisher Scientific, Pittsburgh, PA) for 10 s to break down the cheese clumps and improve the extraction of components and centrifuged at 15,700 × *g* for 3 min at 25°C. The supernatant was employed for further spectroscopic analysis.

### 2.3. FT-IR spectroscopy measurement

The FT-IR spectra of the extractions were collected using a portable FT-IR 4500a (Agilent Technologies, Santa Clara, CA, USA) equipped with a 3-bounce diamond attenuated total reflectance (ATR) accessory. The FT-IR also had Zinc Selenide (ZnSe) beam splitter, a low-powered solid-state laser, a wire-wound element infrared source, and a thermoelectrically cooled deuterated triglycine sulfate (DTGS) detector. The supernatants (10 μL) from the extracts were deposited on the ATR crystal and dried under a vacuum to generate a thin film. The infrared spectra were collected from 4,000 to 650 cm^–1^ with a 4 cm^–1^ resolution, and 64 scans were co-added to increase the signal-to-noise ratio. Four independent spectra were collected from each sample to address the possible heterogeneity of the samples. The means of the four spectra per sample was used for pattern recognition analysis. Thus, a total of 72 spectra were used for model development in this study for the different extraction methods (water-soluble, methanol, and ethanol extracts) obtained from 6(ripening times) x6 (cheese cubes per ripening time) x1 (average of 4 spectra per sample) x2 (cheese production replications).

### 2.4. References analyses

#### 2.4.1. Organic acid determination by high-performance liquid chromatography (HPLC)

The organic acid content of Turkish white cheese samples was determined by extracting the samples with chloroform and distilled water and running the sample through a Sep-Pak C18 Vac solid cartridge (Waters Corp., Milford, MA, USA). The organic acids were determined using an HPLC (1100, Agilent Technologies, Santa Clara, CA, USA), and eluted components were separated through a Prevail organic acid column with dimensions of 150 × 4.6 mm × 5 μm thickness (Hichrom, Berkshire, UK). The elution of the components was carried out isocratically using pH 2.5 phosphate buffer (25 mM KH_2_PO_4_) as a mobile phase at a flow rate of 1.5 mL/min at room temperature. The chromatogram was automatically integrated for the organic acids at 210 nm.

#### 2.4.2. Free amino acid determination by gas chromatography–mass spectrometry (GC-MS)

Free amino acids were extracted by dispersing 100 mg of Turkish white cheese in 1 mL of distilled water through an ultrasonic dismembrator (Fisher Scientific, Pittsburgh, PA, USA) for 10 s; then, it was centrifugated at 15,700 × *g* for 3 min at room temperature. The supernatant was then derivatized using an EZ:faast amino acid analysis kit (KG0-7165, Phenomenex Inc., Torrance, CA, USA). After the derivatization, the sample was injected through a GC (7820A, Agilent Technologies, Santa Clara, CA, USA) coupled with an MS detector (5,977, Agilent Technologies, Santa Clara, CA, USA). The chromatic separation was carried out in a Zebron™ ZB-AAA 10 m × 0.25 mm × 0.25 μm capillary column (Phenomenex^®^, Torrance, CA) with a 0.1 μm film thickness. The MS acquisition was carried out in Scan mode with a range scanned set at 45-450 m/z. After identifying the ions, the quantification was achieved with selected ion monitoring (SIM). After determining the 18 individual free amino acid content in the cheese samples, the total free amino acid content was calculated by summing those 18 free amino acid contents.

#### 2.4.3. Free fatty acid determination by gas chromatography-flame ionization detector (GC-FID)

The fat content of the cheese samples was extracted using a mixture of hexane:methanol (2:1 v/v) and methylated, as explained in Yaman et al. (19). Methylated samples were analyzed through a GC-FID (6890N, Agilent Technologies, Santa Clara, CA, USA). The separation of the fatty acids was achieved in an HP-88 capillary column (100 m × 0.25 mm × 0.2 μm) (Agilent Technologies, Santa Clara, CA, USA). Fatty acids’ identification was verified by comparing the sample peak retention times and percentage with reference standards (Supelco 37 Component FAME Mix, Sigma-Aldrich, St. Louis, MO, USA). The fatty acid concentrations were determined as percent fatty acid. All the reference analyses were performed in duplicate. Short-chain fatty acid content was calculated by adding the content of fatty acids, including Butyric acid, Caproic acid, Caprylic acid, and Capric acid.

### 2.5. Data analysis

The changes in the target components, including individual organic acids, free amino acids, and free fatty acids during the ripening period, were evaluated by descriptive analysis and a randomized ANOVA experimental design with General Linear Model Repeated Measure analysis (SPSS Statistics software version 25.0, IBM Corp., Armonk, NY). The standard error of laboratory (SEL) was calculated according to Berzaghi et al. (20).

Infrared spectral data consist of highly rich as well as complex information (21), and multivariate data analysis was used to extract meaningful information from this complex data set. Cheese sample spectral data were analyzed with multivariate statistical analysis software (Pirouette version 4.5, Infometrix Inc., Bothell, WA, USA). The collected FT-IR spectra were imported as GRAMS (.spc) files and mean-centered, normalized, and smoothed (S-G polynomial fitting algorithm with a 35-point window) prior to the multivariate analysis. Samples with high leverage and/or studentized residual were re-analyzed or labeled as an outlier and excluded from the multivariate model. Soft independent modeling of class analogy (SIMCA) was used to classify the cheese samples according to their ripening days (day 1 to day 100). Partial least squares regression (PLSR) was employed to develop prediction models that quantify biochemical changes in the Turkish white cheese samples. In the regression model, after the outliers were excluded (if any), the remaining data set was subdivided into two groups: calibration/training and external validation. The last-mentioned group was used to unbiasedly estimate the strength of the prediction capabilities (robustness) of the generated training models in real-world situations. After removing the outliers (if any), 80% of the total data set was randomly utilized in the training model, and the remaining 20% was used in the external validation. The validation was generated with cheese samples from any ripening time that came from cheese blocks not used for the calibration set.

The FT-IR spectra of the cheese sample collected over the ripening period were grouped pursuant to the unique biochemical changes that occurred during that period. SIMCA is a supervised classification method that uses known class membership by constructing a multidimensional box for each class (ripening days: class 1: day 1, class 2: day 20… etc.) to generate the model to classify new samples in the future by using an F test (21). The performance of the generated SIMCA models was evaluated through class projection plots, misclassification numbers, and interclass distances (ICD). As a rule of thumb, groups with ICD higher than 3 are accepted as significantly different than each other (22).

The quantification of the specific biochemical compounds in the cheese samples over the ripening period was determined by combining the spectroscopic measurement results with the traditional reference data with chemometric regression approaches. The regression analysis tries to find the best functional relationship between the vector of measured signals (a spectrum in our case) and response (e.g., Lactic acid content) and also finds the optimal value of the parameters that will lead to the lowest error in the prediction of the responses (23). The developed PLSR model’s prediction performance was evaluated using the standard error of cross-validation (SECV), coefficient of determination (r), and outlier diagnostics.

## 3. Results and discussion

### 3.1. Changes in organic acids, fatty acids, and free amino acid concentrations during the ripening period

The starter cultures, *Lc. lactis ssp. cremoris* and *Lc. lactis ssp. lactis*, used for Turkish white cheese production, have been considered critical in developing flavor compounds because of their natural autolytic activity (24–26). Our results (Table 1) showed significant development of organic acids, fatty acids, and free amino acids in cheese during ripening (*P* < 0.05). With respect to organic acids, we found increased levels of lactic, citric, propionic, and acetic acid throughout the ripening period (*P* < 0.05) (Table 1). Citric acid had the highest concentration in terms of the organic acids in the tested cheese samples (Table 1) and showed the most dominant changes during ripening, following a zero-order kinetics (Supplementary Figure 1A) with an average increase in the concentration of 1.40 mg/100 g per day. The high concentration of citric acid was due to the presence of native citrate in milk (∼0.8 mmol/100 mL) (4), besides the production of citrate in the Krebs cycle utilized by lactic acid bacteria (27, 28). The increase in citrate concentration during cheese ripening has been previously reported in white cheese (7). Acetic acid was the second most important organic acid in terms of the formation rate by having a 0.19 mg/100 g increase each day. Acetic acid is generated from different pathways, including generation in the Krebs cycle (acetic acid or acetate formation from acetyl-CoA in the glycolysis step) (11), production from amino acids (glycine, alanine, glutamic acid, and leucine) (29), and lipolysis of fatty acids (4). Even though lactic acid was not predominantly present in the cheese samples, it is the precursor of the main metabolic reactions in cheese; also, it is the main component of numerous aroma compounds in the cheese (4). The limited amount of lactic acid could be due to the removal of most of the lactose (∼98%) in the whey, and the residual lactose (1-2%) in the cheese curd is metabolized to lactic acid by the lactic starter in a short period of time (4). Lastly, propionic acid was the organic acid that had the lowest content in all tested cheese samples throughout the ripening and also increased at a lower rate (0.07 mg/100 g increase each day) than other organic acids with no significant change in propionic acid between days 40 and 60 (Table 1). The propionic acid increase occurs with enzymatic hydrolysis of milk fat and via *Propionibacterium* fermentation. Akalın et al. (7) reported a similar increase in organic acids, particularly lactic, propionic, butyric, and acetic acid, in Turkish white cheese when using a starter culture that consisted of equal rates of *Lactococcus lactis* ssp. cremoris and *Lactococcus lactis* ssp. Lactis.

**TABLE 1:** Changes in certain organic acids, free fatty acids, and free amino acids in white cheese during the ripening period (Mean ± SD).

Compound	Units	Ripening day						
		1	20	40	60	80	100	SEL
Lactic	mg/100 g cheese	13.77 ± 0.49^a^	14.78 ± 0.54^b^	15.82 ± 0.39^c^	17.56 ± 0.43^d^	19.84 ± 0.48^e^	24.21 ± 0.59^f^	0.39
Citric		132.96 ± 3.23^a^	164.8 ± 6.00^b^	184.82 ± 8.55^c^	205.16 ± 9.49^d^	231.82 ± 10.72^e^	282.83 ± 13.07^f^	3.60
Propionic		7.96 ± 0.29^a^	9.9 ± 0.53^b^	11.10 ± 0.46^c^	11.07 ± 0.55^c^	13.00 ± 0.52^d^	15.38 ± 0.82^e^	0.28
Acetic		20.16 ± 0.94^a^	22.38 ± 1.04^b^	25.28 ± 1.18^c^	28.25 ± 0.95^d^	34.46 ± 1.15^e^	38.45 ± 0.93^f^	0.87
SCFA (C_4_ – C_10_)		5.38 ± 0.33^a^	7.26 ± 0.61^b^	8.75 ± 0.87^c^	10.23 ± 0.64^d^	11.62 ± 0.52^e^	12.45 ± 0.47^f^	0.38
Lauric acid (C_12_)		5.63 ± 0.23^a^	7.72 ± 0.39^b^	6.98 ± 0.33^c^	7.01 ± 0.36^c^	7.82 ± 0.27^b^	8.14 ± 0.28^d^	0.24
Myristic acid (C_14_)		19.61 ± 0.42^a^	25.97 ± 0.44^b^	24.74 ± 0.86^c^	24.36 ± 0.34^d^	24.11 ± 0.79^d^	25.09 ± 0.34^e^	0.40
Palmitic acid (C_16_)		63.74 ± 0.71^a^	79.51 ± 2.9^b^	76.78 ± 1.52^c^	77.21 ± 1.03^c^	78.43 ± 1.62^d^	80.57 ± 1.81^e^	1.08
Stearic acid (C_18_)		25.6 ± 0.27^a^	31.55 ± 0.51^b^	29.96 ± 1.04^c^	30.28 ± 0.56^c^	30.82 ± 0.71^d^	31.97 ± 0.62^e^	0.35
Oleic acid (C_18:1_)		35.75 ± 1.15^a^	45.59 ± 0.98^b^	42.61 ± 0.73^c^	42.08 ± 1.32^c^	43.55 ± 0.52^d^	45.64 ± 0.62^b^	0.79
Linoleic acid (C_18:2_)		5.70 ± 0.17^a^	7.10 ± 0.32^b^	6.93 ± 0.12^c^	6.81 ± 0.27^c^	7.39 ± 0.16^d^	7.47 ± 0.22^d^	0.16
Glycine	μg/100 g cheese	10.11 ± 0.16^a^	10.03 ± 0.05^b^	10.35 ± 0.05^c^	10.66 ± 0.06^d^	10.93 ± 0.06^e^	11.15 ± 0.06^f^	0.06
Alanine		18.55 ± 0.09^a^	18.69 ± 0.09^b^	19.24 ± 0.1^c^	19.78 ± 0.11^d^	20.28 ± 0.12^e^	20.68 ± 0.10^f^	0.07
Valine		9.5 ± 0.05^a^	9.64 ± 0.05^b^	9.9 ± 0.05^c^	10.16 ± 0.06^d^	10.41 ± 0.06^e^	10.61 ± 0.05^f^	0.03
Isoleucine		6.31 ± 0.03^a^	6.45 ± 0.03^b^	6.61 ± 0.03^c^	6.77 ± 0.04^d^	6.94 ± 0.04^e^	7.07 ± 0.04^f^	0.02
Methionine		11.57 ± 0.06^a^	11.89 ± 0.06^b^	12.16 ± 0.06^c^	12.44 ± 0.07^d^	12.74 ± 0.07^e^	12.97 ± 0.07^f^	0.04
Proline		23.24 ± 0.12^a^	24.04 ± 0.12^b^	24.54 ± 0.13^c^	25.07 ± 0.14^d^	25.65 ± 0.15^e^	26.12 ± 0.13^f^	0.08
Phenylalanine		14.47 ± 0.07^a^	15.06 ± 0.07^b^	15.35 ± 0.08^c^	15.65 ± 0.09^d^	16.01 ± 0.09^e^	16.3 ± 0.08^f^	0.05
Tyrosine		21.77 ± 0.11^a^	22.81 ± 0.11^b^	23.2 ± 0.12^c^	23.62 ± 0.13^d^	24.15 ± 0.14^e^	24.57 ± 0.13^f^	0.08
Tryptophan		43.89 ± 0.22^a^	46.28 ± 0.23^b^	46.97 ± 0.24^c^	47.76 ± 0.26^d^	48.8 ± 0.28^e^	49.65 ± 0.25^f^	0.16
Serine		73.41 ± 0.37^a^	73.49 ± 0.36^a^	75.81 ± 0.39^b^	78.04 ± 0.43^c^	80.07 ± 0.46^d^	81.65 ± 0.41^e^	0.26
Threonine		13.87 ± 0.07^a^	13.98 ± 0.07^b^	14.39 ± 0.08^c^	14.79 ± 0.08^d^	15.17 ± 0.09^e^	15.46 ± 0.08^e^	0.05
Cysteine		1.27 ± 0.03^a^	1.29 ± 0.02^b^	1.32 ± 0.02^c^	1.36 ± 0.05^d^	1.39 ± 0.02^e^	1.42 ± 0.02^e^	0.00
Lysine		41.81 ± 0.21^a^	42.69 ± 0.21^b^	43.77 ± 0.22^c^	44.84 ± 0.25^d^	45.94 ± 0.27^e^	46.8 ± 0.24^e^	0.15
Histidine		30.87 ± 0.15^a^	31.73 ± 0.16^b^	32.46 ± 0.16^c^	33.21 ± 0.18^d^	34.00 ± 0.20^e^	34.63 ± 0.18^e^	0.11
Aspartic acid		8.36 ± 0.04^a^	8.65 ± 0.04^b^	8.83 ± 0.05^c^	9.02 ± 0.05^d^	9.23 ± 0.05^e^	9.40 ± 0.05^e^	0.03
Glutamic acid		31.03 ± 0.15^a^	32.3 ± 0.16^b^	32.92 ± 0.17^c^	33.57 ± 0.19^d^	34.33 ± 0.20^e^	34.95 ± 0.18^e^	0.11
Asparagine		35.45 ± 0.18^a^	37.15 ± 0.19^b^	37.78 ± 0.19^c^	38.46 ± 0.21^d^	39.32 ± 0.23^e^	40.02 ± 0.20^e^	0.13
Glutamine		60.50 ± 0.30^a^	63.8 ± 0.32^b^	64.75 ± 0.33^c^	65.84 ± 0.36^d^	67.28 ± 0.39^e^	68.44 ± 0.35^e^	0.22
TFAA		455.98 ± 2.36^a^	469.97 ± 2.33^b^	480.36 ± 2.46^c^	491.04 ± 2.70^d^	502.65 ± 2.91^e^	511.89 ± 2.58^f^	1.66

^a–f^Different superscript in the same line indicates significant differences (*p* < 0.05); SCFA: short chain fatty acids; TFAA: Total free amino acid; SD: standard deviation; SEL: standard error of laboratory.

The free fatty acids (FFA) in cheese are produced by lipolytic processes (C_4_–C_18:1_) and bacterial fermentation (C_2_–C_4_). The FFA contents in the samples are presented in Table 1. Myristic, stearic, palmitic, and oleic acids contents all showed similar trends by increasing rapidly during the first 20-days of ripening, and then they all reached a plateau. On the other hand, the SCFAs accumulated linearly throughout the ripening period by showing a zero-order kinetic (Supplementary Figure 1B). Instead, the hydrolysis of lauric and linoleic acids were flat throughout the ripening (Supplementary Figure 1B). Akin and others (30) also reported similar FFA results in white cheese ripening using the same lactic acid starter bacteria. The plateau and the limited increase could be explained by the transesterification reactions between alcohol and fatty acids throughout ripening that result in fatty acid esters formation (31). Therefore, increasing FFA content through lipolysis was restricted in our samples.

Finally, the free amino acid levels of the cheese samples (Table 1) increased throughout the ripening period (*P* < 0.05) (Supplementary Figures 1C, D). Total free amino acids (TFAAs) change linearly over time with a rate of production (0.714 μg/100 g increase per day). The leading free amino acid produced was serine (0.091 μg/100 g), followed by glutamine (0.074 μg/100 g), tryptophane (0.053 μg/100 g), lysine (0.051 μg/100 g), asparagine (0.043 μg/100 g), histidine (0.038 μg/100 g), and glutamic acid (0.038 μg/100 g). The other free amino acids showed lower production rates (average of 0.017 μg/100 g increase per day). The production of free amino acids during the ripening of Turkish white cheese was low compared to organic acids and fatty acids, likely associated with the starters’ modest proteolytic characteristics.

### 3.2. FT-IR spectra to monitor biochemical changes during cheese ripening

**FIGURE 1:**
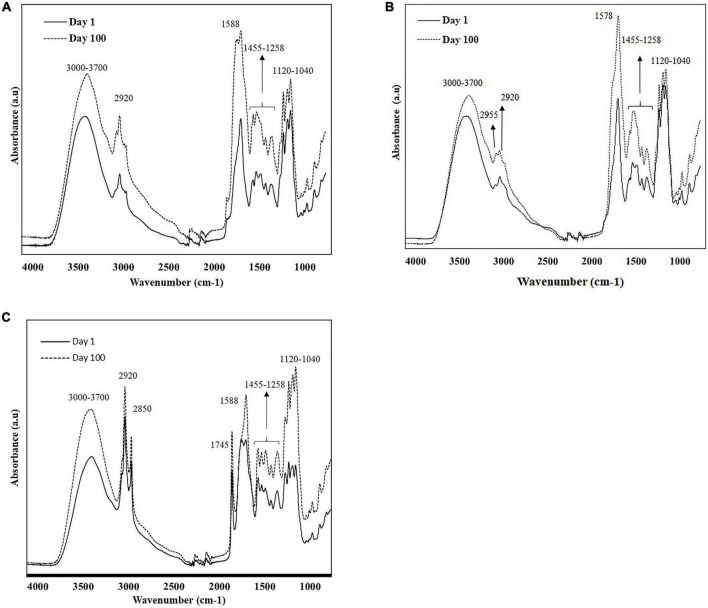
Characteristic raw spectra of soluble extracts of cheese samples **(A)** water; **(B)** methanol; and **(C)** ethanol on day 1 and 100, were collected using portable FT-IR (4,000-650 cm^–1^).

Figures 1A–C show the overlaid FT-IR spectra (4,000 – 650 cm^–1^) of different extracts of cheese samples on days 1 and 100, exhibiting noticeable changes during the ripening process, primarily in the spectral range 1,800-900 cm^–1^. The spectra collected from the different extracts showed unique patterns depending on the solubility of the cheese component in the solvents (Figures 1A–C).

The broad band at 3,700 to 3,000 cm^–1^ was associated with O-H stretching vibrations of hydroxyl groups (water); while the bands centered at 2,955, 2,920, and 2,850 cm^–1^ were related to methylene (CH_3_) asymmetrical and symmetrical stretching and methyl (CH_2_) asymmetrical stretching of lipids (32–35). The distinct band at 1,745 cm^–1^ corresponds to the ester carbonyl (–C = O) functional group of lipids (33, 36). The prominent band at 1,578 cm^–1^ and its left shoulder (1,645 cm^–1^) were attributed to the amide groups in proteins, peptides, and free amino acids (36–38). The shoulder band at 1,453 cm^–1^ corresponded to methylene bending of amino side chains and the CH_3_ asymmetric deformation of amines (33, 38, 39). The band at aground 1,425 cm^–1^ was related to acidic amino acids (COO^–^ symmetric stretching) (38), while the bands between 1,390 and 1,429 cm^–1^ can be related to the [–CH(CH_3_)] groups in amino acids such as alanine, valine, leucine, isoleucine, and methionine. The band at 1,315 cm^–1^ was responsible for the plane bending vibration of CH bonds of amino acids (40). The band at 1,159 cm^–1^ corresponded to ester linkages of fats (C-O) (41). The bands between 1,120 and 1,040 cm^–1^ were associated to C-O stretching and OH bending groups of organic acids and residual lactose (38). Cheese ripening from day 1 to day 100 showed an increase in the intensity of the infrared signal in all extracts (Figures 1A–C), evidencing the extent of proteolysis, lipolysis, and lactose metabolism during white cheese maturation.

Even though the overall spectra of the different cheese extracts (water, methanol, and ethanol) showed similar spectral characteristics, there were some remarkable distinctions due to the solvent polarity (Figures 1A–C). The region related to fats (2,955-2,850 cm^–1^, 1,745 cm^–1^ and 1,159 cm^–1^) showed higher intensities for ethanol extracts showing its ability to penetrate through the cell membrane and dissolve hydrophobic chains compared to methanol (42). Water and methanol extracts showed more intense signal in the 1,650-1,570 cm^–1^, attributed to protein bands, compared to ethanol extracts. The solubilities of the amino acids in water are high because they are present predominantly in zwitterionic form and their solubility decrease with the increase in the hydrophobic character of the solvents (43).

**FIGURE 2:**
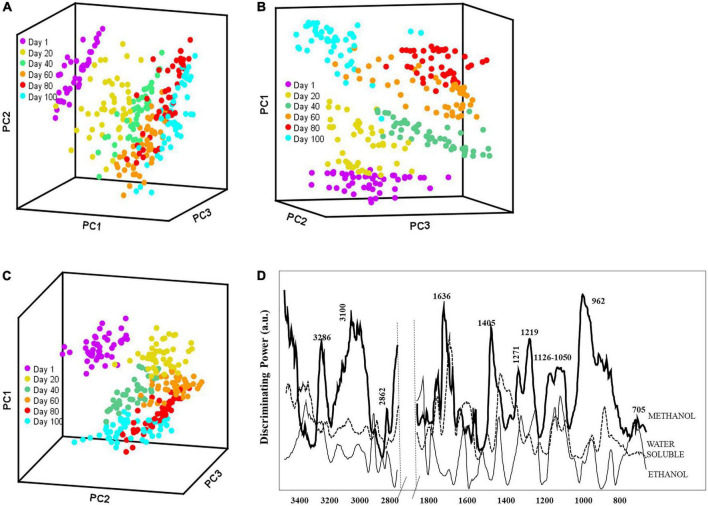
Soft independent modeling of class analogy (SIMCA) 3D projection plots for spectra collected by portable FT-IR spectrometer for soluble extracts **(A)** water; **(B)** methanol; and **(C)** ethanol for 100 days ripening period and **(D)** SIMCA discriminating plot showing the bands and regions responsible for class separation based on the corresponding FT-IR SIMCA model.

The FT-IR spectra were analyzed using pattern recognition analysis (SIMCA) to evaluate the effects of solvent extracts to classify cheese ripening stages (Figures 2A–C). The boundaries around the samples define the regions in which samples belong to a specific class fall within 95% confidence (44). The projection plots for all extraction methods showed distinct groups, with most interclass distances (ICDs) above 3, indicating that the different solvents extracted cheese metabolites that allowed to classify samples during the complex process of ripening (Tables 2A–C). In general, the greater the ICDs between two clusters, the more chemically distinct. The diagonal values of the ICDs decreased with ripening time, indicating smaller biochemical changes as cheese aging increases. Methanol extracts showed the largest ICDs between clusters indicating that its mixed polarity allowed to solubilize a wider array of metabolites compared to water and ethanol.

**TABLE 2:** Interclass distances between various cheese extracts (a: water; b: methanol; and c: ethanol) on different ripening ages, based on SIMCA model generated by the FT-IR spectral data.

(A)						
Ripening day	1	20	40	60	80	100
1	0.0					
20	7.3^a^	0.0				
40	14.2	3.6^b^	0.0			
60	16.5	4.9	4.2^c^	0.0		
80	15.8	6.3	4.3	3.2^d^	0.0	
100	15.4	7.1	4.6	3.3	1.5^e^	0.0
**(B)**						
**Ripening day**	**1**	**20**	**40**	**60**	**80**	**100**
1	0.0					
20	14.6^a^	0.0				
40	24.5	9.5^b^	0.0			
60	29.9	15.2	11.4^c^	0.0		
80	34.5	14.8	13.2	7.7^d^	0.0	
100	20.7	8.9	8.2	5.9	4.3^e^	0.0
**(C)**						
**Ripening day**	**1**	**20**	**40**	**60**	**80**	**100**
1	0.0					
20	5.3^a^	0.0				
40	8.5	6.2^b^	0.0			
60	11.3	8.6	4.6^c^	0.0		
80	11.0	9.8	4.7	3.4^d^	0.0	
100	13.6	14.9	7.1	7.3	2.7^e^	0.0

^a^Stage 1 (day 1 to 20), ^b^Stage 2 (day 20 to 40), ^c^Stage 3 (day 40 to 60), ^d^Stage 4 (day 60 to 80), ^e^Stage 5 (day 80 to 100).

We evaluated the discriminating power plots in SIMCA that shows the variables with a predominant effect on the sample grouping. Model developed with water-based extracts employed 3 factors to explain 97.8% of the variance in the data set. It is important to highlight that water extracts were further fractionated with chloroform (45), resulting in negligible amounts of neutral fat in the samples. The discriminating power (Figure 2D) for water extracts showed that the spectral regions from 1,680 to 1,510, 1,430 to 1,360, and 1,100 to 1,040 cm^–1^ were important in classifying Turkish white cheese samples based on the ripening process. The bands between 1,680 and 1,510 cm^–1^ comprise the absorbances from amide functional groups (46), and the bands between 1,430 to 1,360 cm^–1^ were related to amide III (47, 48). Furthermore, the smaller bands at 1,093 – 1,041 and 855 cm^–1^ could correlate with the organic acids in the white cheese. The changes in the amino acids and organic acids though out the ripening period (Table 1) also support these findings. Methanol extracts also employed 3 factors and explained 98.2% of the variance. The discriminating power plot revealed that bands at around 3,290 to 2,880, 1,636 to 1,611, 1,405, and 1,219 to 960 cm^–1^ were responsible for the class separations (Figure 2D). Methanol is a polar organic solvent that is effective in dissolving amino (1,650 – 1,219 cm^–1^) and organic (1,096 – 962 cm^–1^) acids but can also solubilize some non-polar compounds such as fatty acids (3,290 – 2,880 cm^–1^). Finally, the discriminating power plot for ethanol extracts revealed that the clustering between classes was mainly related to the regions between 3,080 – 2,850 cm^–1^ and 1,700 – 1,685 cm^–1^ (Figure 2D), which can be corresponded to the-C-H symmetric and asymmetric stretching of fatty acids and fatty acids’ esters, respectively (19, 45). The band at around 1,400 cm^–1^ can be associated with acidic amino acids and the aliphatic chains of fatty acids (45). As a polar organic solvent, ethanol also had an affinity for amino and organic acids, indicated by the signal in the range of 1,230 – 835 cm^–1^.

### 3.3. PLSR models for the prediction of individual organic acids, free fatty acids, and total free amino acids

Individual organic acids, free fatty acids, and free amino acids content in Turkish white cheese samples were correlated with the FT-IR spectra of the various extraction methods (including water-based solution, methanol, and ethanol) using PLSR algorithms to generate prediction models. As mentioned earlier, the sample set was divided into the calibration/training and external validation sets, and the robustness/strength of the generated calibration models was tested using the external validation set. The performance statistics of each model, as well as the range of the values and the number of samples included in each model, were given in Tables 3, 4. In order to improve the predictive capacity of each model, mathematical pre-processing procedures were employed, and the frequency regions that give low regression coefficients were excluded from each model since those regions were dominated by noisy, unreliable variables (49). The specific bands and ranges for the model generation were shown in Supplementary Table 1.

**TABLE 3:** Performance statistics of the calibration models developed using FT-IR, for the prediction of free amino acids, organic acids, and free fatty acids in soluble extracts (water, methanol, and ethanol) samples during the ripening period.

Compound	Level range (mg/100g)	Water				Methanol				Ethanol			
		N^c^	F^d^	SECV^e^	R_CV_^f^	N	F	SECV	R_CV_	N	F	SECV	R_CV_
Lactic acid	12.6–25.2	55	6	0.88	0.97	54	6	0.96	0.95	55	6	0.99	0.96
Citric acid	128.7–295.8	57	6	10.65	0.98	55	6	11.35	0.97	55	6	15.98	0.94
Propionic	7.41–16.3	58	4	0.71	0.95	58	4	0.72	0.95	55	4	0.71	0.95
Acetic acid	18.0–39.6	56	5	1.28	0.98	56	6	1.52	0.97	55	6	1.99	0.95
SCFA^a^	4.7–14.4	56	5	0.64	0.97	55	6	0.51	0.98	56	5	0.51	0.98
Lauric acid	4.9–9.0	55	5	0.58	0.92	55	5	0.48	0.93	55	5	0.45	0.94
Myristic acid	17.8–27.2	54	6	0.83	0.92	55	6	0.80	0.93	56	5	0.72	0.94
Palmitic acid	58.8–89.6	55	6	2.20	0.93	56	6	2.09	0.94	56	6	1.85	0.95
Stearic acid	23.8–34.8	55	5	0.93	0.91	55	6	0.82	0.93	55	6	0.79	0.93
Oleic acid	32.0–49.2	54	6	1.34	0.92	55	5	1.09	0.94	56	4	1.01	0.94
Linoleic acid	5.13–8.2	55	6	0.24	0.93	55	6	0.24	0.93	54	6	0.23	0.93
TFAA^b^	0.58–0.66	55	4	0.0029	0.99	55	4	0.0039	0.98	54	5	0.0048	0.96

^a^Short chain fatty acids, ^b^Total free amino acid, ^c^Number of samples used in calibration models, ^d^Number of factors (latent variables) used to generate the models, ^e^Standard error of cross validation, ^f^Correlation coefficient of cross-validation. For each model 58 sample were exist before excluding the outliers.

**TABLE 4:** Performance statistics of the validation models developed using FT-IR, for the prediction of free amino acids, organic acids, and free fatty acids in soluble extracts (water, methanol, and ethanol) samples during the ripening period.

Compound	Level range (mg/100g)	Water			Methanol			Ethanol		
		N^c^	SEP^d^	R_Pre_^e^	N	SEP	R_Pre_	N	SEP	R_Pre_
Lactic acid	13.5–25.2	14	1.12	0.97	14	1.00	0.96	14	1.10	0.95
Citric acid	128.7–299.7	14	10.51	0.98	14	12.80	0.96	14	13.18	0.96
Propionic	7.4–16.3	14	0.57	0.97	14	0.87	0.92	14	0.64	0.96
Acetic acid	19.8–38.4	14	1.67	0.97	14	1.78	0.96	14	2.19	0.94
SCFA^a^	4.7–12.9	14	0.72	0.97	14	0.67	0.96	14	0.41	0.99
Lauric acid	4.9–9.0	14	0.63	0.92	14	0.52	0.93	14	0.38	0.95
Myristic acid	18.0–27.1	14	0.79	0.93	14	0.82	0.93	14	0.80	0.93
Palmitic acid	59.0–88.0	14	2.00	0.92	14	1.81	0.94	14	1.95	0.95
Stearic acid	23.8–34.6	14	0.92	0.92	14	0.72	0.95	14	1.07	0.94
Oleic acid	32.6–49.0	14	1.53	0.93	14	1.18	0.95	14	0.95	0.96
Linoleic acid	5.1–8.2	14	0.24	0.91	14	0.23	0.92	14	0.19	0.94
TFAA^b^	0.58–0.66	14	0.0037	0.99	14	0.0040	0.98	14	0.0057	0.95

^a^Short chain fatty acids, ^b^Total free amino acid, ^c^Number of samples used in calibration models, ^d^Standard error of prediction, ^f^Correlation coefficient of prediction for validation.

Depending on the target compound and extraction method, the cross-validation (leave-one-out) approach determined two to six LVs to generate the models (Table 3), and those LVs explained 95.36 to 99.96% of the variance in the generated calibration models. The regression models for different extraction methods showed a strong correlation (0.98 ≥ R_CV_ ≥ 0.91 and 0.99 ≥ R_Pre_ ≥ 0.91) in predicting individual organic acids, free fatty acids, and total free amino acids (Tables 3, 4). The standard error of prediction (SEP) values ranged from 0.57 to 13.18 mg/100 g cheese for individual organic acids, 0.19 to 2.00 mg/100g cheese for SCFA and individual free fatty acids, and 0.0037 to 0.0057 mg/100 g cheese for total free amino acids; the numbers were similar to the standard error of cross-validation (SECV) for their corresponding models (Tables 3, 4) that indicates the good predictive ability of the generated calibration models. Even though all types of extraction methods provided similar prediction performances, the organic and amino acids were predicted slightly better with the water-based solution, while free fatty acids levels were predicted best in ethanol-extracted samples.

## 4. Conclusion

This study proposes how to improve the FT-IR prediction performance by using simple extraction methods using various solvents or mixtures to monitor soluble further ripening compounds such as free amino acids, organic acids, and fatty acids that occur with the biochemical reactions in cheese during ripening. We found that ethanol was more effective than methanol in extracting free fatty acids providing the best measurement performance for this class of compounds. However, water-based extracts were most effective in solubilizing organic acids and free amino acids. The mixed polarity of methanol provided the best classification of metabolites during ripening. Selection of extraction solvent (water, methanol, ethanol) had a slight effect on the ability of FT-IR to monitor cheese ripening and predict several cheese components. This study could help cheese manufacturers easily monitor the rate and the products of biochemical reactions, including lipolysis, proteolysis, and glycolysis, that produce essential flavor and texture characteristics in white cheese.

## Data availability statement

The raw data supporting the conclusions of this article will be made available by the authors, without undue reservation.

## Author contributions

HY: methodology, validation, formal analysis, investigation, writing—original draft, and visualization. DA: methodology, validation, formal analysis, investigation, writing— original draft, and visualization. LR-S: conceptualization, validation, resources, writing—review and editing, and supervision. All authors contributed to the article and approved the submitted version.
