# Supplementary figures and images for: Monitoring Turkish white cheese ripening by portable FT-IR spectroscopy
Source: Front Nutr. 2023 Feb 6;10:1107491. doi: 10.3389/fnut.2023.1107491 (PMC9940898; doi:10.3389/fnut.2023.1107491)

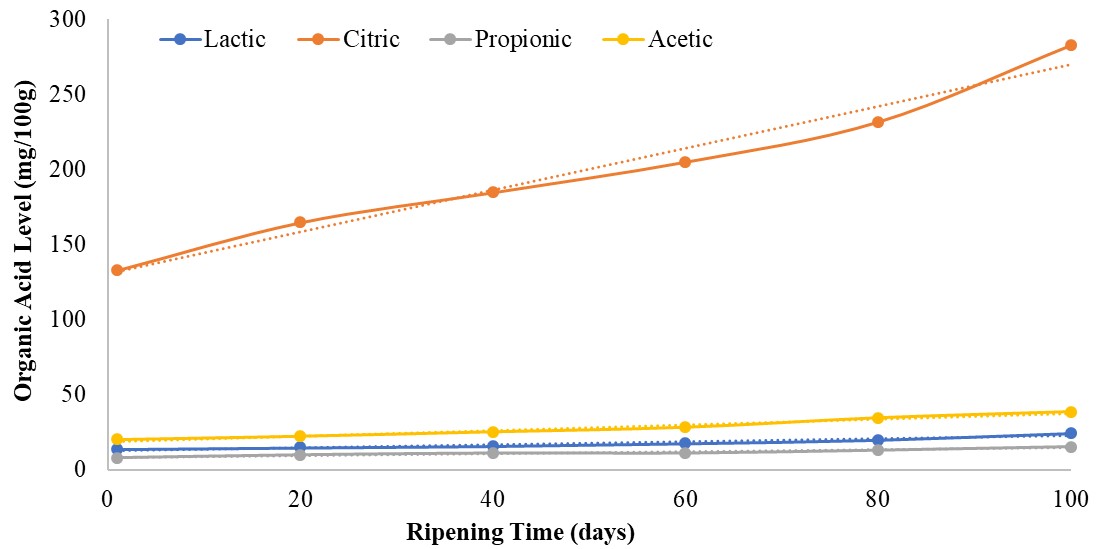

Supplement: Supplementary file 1 [file Data_Sheet_1.zip › Supplementary Figures/Supplementary Figures 1A.JPEG]

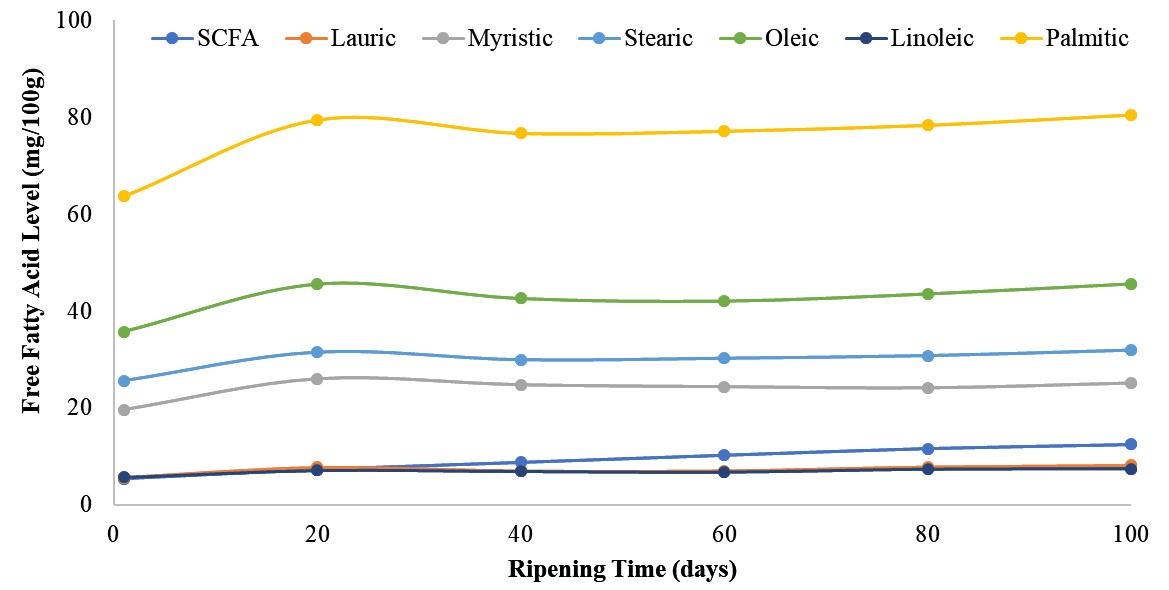

Supplement: Supplementary file 1 [file Data_Sheet_1.zip › Supplementary Figures/Supplementary Figures 1B.JPEG]

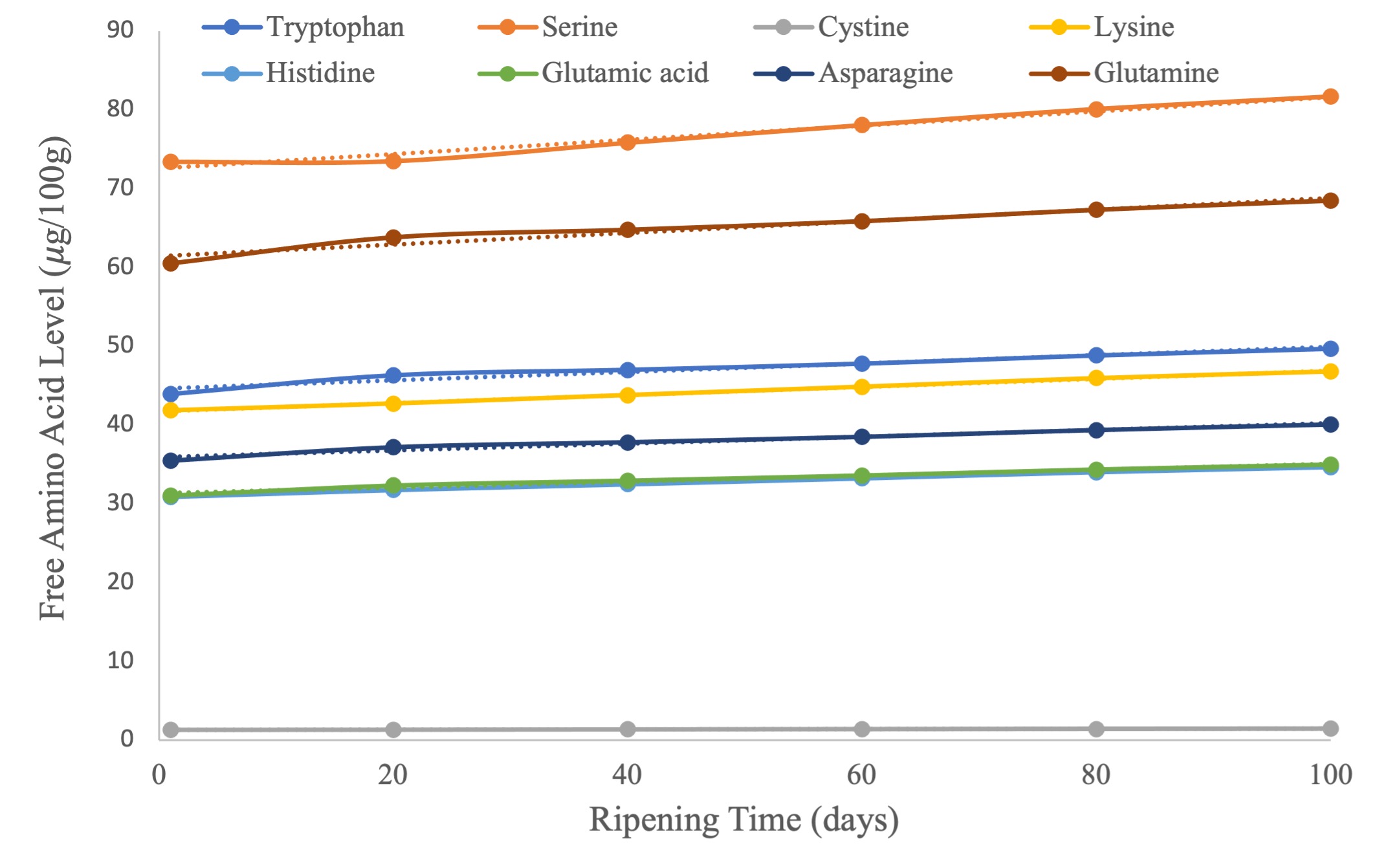

Supplement: Supplementary file 1 [file Data_Sheet_1.zip › Supplementary Figures/Supplementary Figures 1C.JPEG]

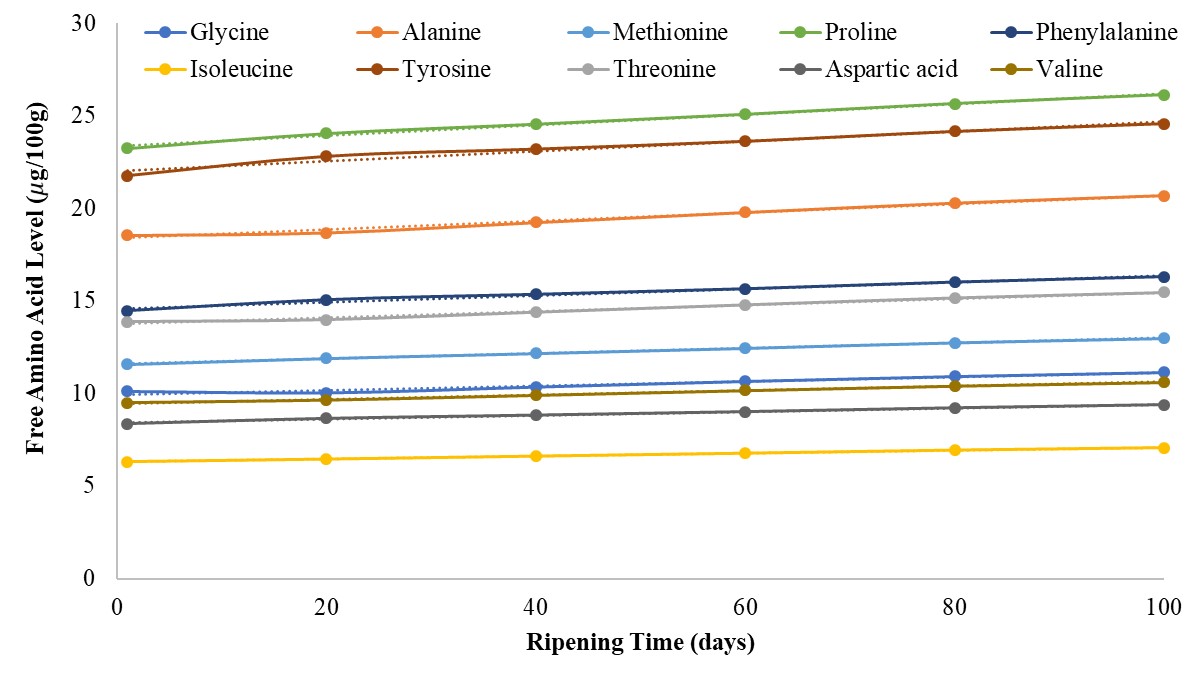

Supplement: Supplementary file 1 [file Data_Sheet_1.zip › Supplementary Figures/Supplementary Figures 1D.JPEG]
