# Supplementary material for: Monitoring Turkish white cheese ripening by portable FT-IR spectroscopy
Source: Front Nutr. 2023 Feb 6;10:1107491. doi: 10.3389/fnut.2023.1107491 (PMC9940898; doi:10.3389/fnut.2023.1107491)
Supplement: Supplementary file 2 [file Table_1.docx]

**SUPPLEMENTARY DATA**

**Title**

Monitoring Turkish White Cheese Ripening by Portable FT-IR Spectroscopy

**Authors**

Hulya Yaman^1,2^, Didem P Aykas^1,3^, Luis E Rodriguez-Saona^1*^

**Affiliation of the authors**

^1^ Department of Food Science and Technology, The Ohio State University, 2015 Fyffe Road, Columbus, Ohio, 43210, USA

^2^ Department of Food Processing, Bolu Abant Izzet Baysal University, Bolu, Turkey

^3^Department of Food Engineering, Adnan Menderes University, Aydin, 09100, Turkey

**Supplementary Table 1.** Band assignments of the side chain and/or specific bands, and ranges that was used for particular amino acids, organic acids, and fatty acids used for the PLSR model generation.

| ***Amino acids*** | ***Band position/cm^−1^*** | ***Assignments*** | ***References*** | ***Skeletal formula*** |
| --- | --- | --- | --- | --- |
| Alanine | 1470,1464,1455  1378  1340  1290 | δas (CH3)  δs (CH3)  δ (CH)  γ(CH2) | Colthup et al. (1975)  Wright and Vanderkooi (1997)  Chirgadze et al. (1975)  Barth (2000) | \| 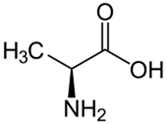 \| \| --- \| |
| Asparagine | 1680  1612–1622  1402 | ν(C=O)  δ (NH2)  νs(COO-) | Pinchas and Laulicht (1971)  Chirgadze et al. (1975)  Venyaminov and Kalnin (1990)  Barth (2000) | 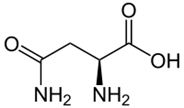 |
| Aspartic Acid | 1716–1788  1574–1579  1402-1404  1264–1450  1120-1250 | ν(C=O)  νas(COO-)  νs(COO-)  δ (COH)  ν(C=O) | Pinchas and Laulicht (1971)  Chirgadze et al. (1975)  Venyaminov and Kalnin (1990)  Rahmelow et al. (1998)  Barth (2000)  Sengupta and Krimm (1985) | 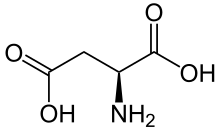 |
| Cystine | 2551, 1849  1424-1432  1296-1303 | ν(SH)  δ(CH2)  γ(CH2) | Susi et al. (1983)  Colthup et al. (1975)  Lewis and McElhaney (1996)  Barth (2000) | \| 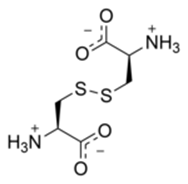 \| \| --- \| |
| Glutamic Acid | 1788-1775  1556-1527  1402  1264–1450  1120-125 | ν(C=O)  νas(COO-)  νs(COO-)  δ (COH)  ν(C=O) | Pinchas and Laulicht (1971)  Chirgadze et al.(1975)  Venyaminov and Kalnin (1990)  Sengupta and Krimm (1985)  Barth (2000) | 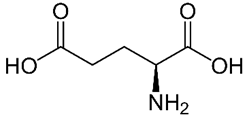 |
| Glutamine | 1704  1612–1622  1556–1560  1404 | ν(C=O)  δ (NH2)  νas(COO-)  νs(COO-) | Pinchas and Laulicht (1971)  Chirgadze et al. (1975)  Venyaminov and Kalnin (1990)  Barth (2000) | 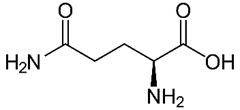 |
| Glycine | 1668–1687  1586–1610  1410 | ν(C=O)  δ(NH2)  ν(CN) | Chirgadze et al. (1975)  Venyaminov and Kalnin (1990)  Dhamelincourt and Ramirez (1993)  Rahmelow et al. (1998)  Barth (2000) | 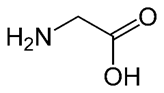 |
| Histidine | 1631  1575,1594  1439  1217, 1229, 1199  1104,1090,1106,1094 | ν(C=C)  ν(C=C)  δ (CH3), ν(CN)  δ (CH), ν(CN), δ (NH2)  δ (CH), ν(NC) | Chirgadze et al. (1975)  Hienerwadel et al. (1997)  Noguchi et al. (1999)  Hasegawa et al. (2000)  Venyaminov and Kalnin (1990)  Barth (2000) | 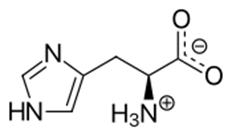 |
| Isoleucine | 1445 1465 1320 | δas (CH3) δ (CH2) δ (CH) | Colthup et al. (1975) Lewis and McElhaney (1996) Barth (2000) | \| 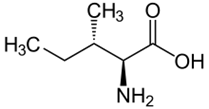 \| \| --- \| |
| Lysine | 1626–1629 1526–1527 | δas(NH3+) δs(NH3+) | Pinchas and Laulicht (1971) Venyaminov and Kalnin (1990) Rahmelow et al. (1998) Barth (2000) | 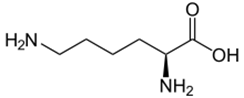 |
| Methionine | 1277 1227 2922 | δ (CH3) δ( S-CH), (C-CH) δas (CH2) | Ramachandran and Natarajan (2006) | 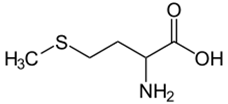 |
| Phenylalanine | 1605 1585 1494 | ν(CC ring) ν(CC ring) ν(CC ring) | Colthup et al. (1975) Venyaminov and Kalnin (1990) Chirgadze et al. (1975) Berendzen and Braunstein (1990) Fabian et al. (1996) | 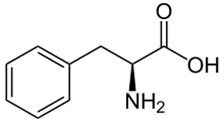 |
| Proline | 1400–1465 1375, 1317 1290 | ν(CN) δ (CH) γ(CH2) | Caswell and Spiro (1987) Rothschild et al. (1989) Gerwert et al. (1990) (Colthup et al., 1975) | 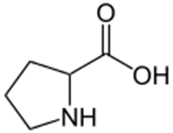 |
| Serine | 1181–1420 1030 983 | δ(COH) or δ(CO2H), ν(CO) ν(C-O) | Pinchas and Laulicht (1971) Colthup et al. (1975) Madec et al. (1978) Susi et al. (1983) | 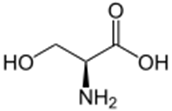 |
| Threonine | 1075–1150 865–942 | ν(C-O) δ(CO2H) | Colthup et al. (1975) Pinchas and Laulicht (1971) | 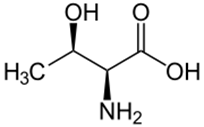 |
| Tryptophan | 1622 1509 1496 1462 1412–1435 1245 | ν(CC), ν(C=C) ν(CN), δ(CH),δ(NH) ν(CC), δ(CH) δ(CH), ν(CC), ν(CN) δ(NH), ν(CC), δ(CH) δ(CH), ν(CC) | Takeuchi and Harada (1986) Lautie´ et al. (1980) Lagant et al. (1998) | 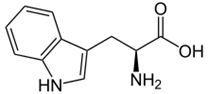 |
| Tyrosine | 1614–1621  1599–1602  1516–1518  1498–1500  1269–1273  1235–1270  1169–1260 | ν(CC), δ(CH)  ν(CC)  ν(CC), δ(CH)  ν(CC), δ(CH)  ν(CC), δ(CH)  ν(CC), δ(CO)  δ(COH) | Chirgadze et al. (1975)  Dollinger et al. (1986)  Takeuchi et al. (1988)  Venyaminov and Kalnin (1990)  Hienerwadel et al. (1997)  Rahmelow et al. (1998)  Chirgadze et al. (1975)  Rothschild et al. (1986) | 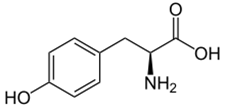 |
| Valine | 1460  1355, 1320 | δas(CH3)  δ(CH) | Overman and Thomas (1999) | \| 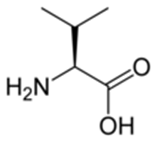 \| \| --- \| |
| ***Organic Acids*** | ***Band position/cm^−1^*** | ***Assignments*** | ***References*** | ***Skeletal formula*** |
| Acetic acid | 3240  3144  1148  1175  995  956 | νs(OH)  γs(OH)  γ(CH3)  νs(CO)  βs(OH)  νs(CO) | Petrovic et al. (2009)  Chen et al., (1998)  Koca et al. (2007) | 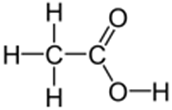 |
| Citric acid | 1721  1105  778 | COO-  δ(COH)  γ(CH2) | Chen et al., (1998)  Koca et al. (2007) | \| 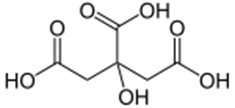 \| \| --- \| |
| Lactic acid | 1730  1211  1124  1095 | COO-  δ(CH)  δ(CO)  δ(CH) | Paucean et al. (2017)  Chen et al., (1998)  Koca et al. (2007) | 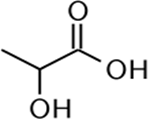 |
| Propionic acid | 1706  1075-1078  816  1231 | C=O  δ(CO)  δ(C-O)  δ(COH) | Umadevi and Thomas (2010)  Chen et al., (1998)  Koca et al. (2007) | 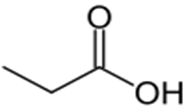 |
| ***Free Fatty Acids*** | ***Band position/cm^−1^*** | ***Assignments*** | ***References*** | ***Skeletal formula*** |
| Butyric acid (C4) | 1745  1460  1175  1416  1280-1220  938 | COO-  δ(CH)  δ(CO)  δ (COH)  νs(CO)  δ(OH | Chen et al., (1998)  Koca et al. (2007)  Aykas and Rodriguez-Saona (2016) | 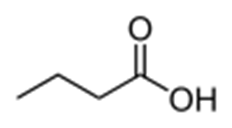 |
| Caproic acid (C6) | 1415  1745  1460  1175  940-1030 | δ(COH)  COO-  δ(CH)  δ(CO)  δ(OH | Chen et al., (1998)  Koca et al. (2007)  Aykas and Rodriguez-Saona (2016) | 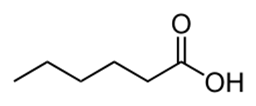 |
| Caprylic acid (C8) | 1745  1460  1175  940-1030 | COO-  δ(CH)  δ(CO)  δ(OH | Chen et al., (1998)  Koca et al. (2007)  Aykas and Rodriguez-Saona (2016) | 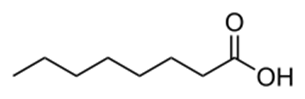 |
| Capric acid (C10) | 1745  1460  1175  940-1030 | COO-  δ(CH)  δ(CO)  δ(CH),δ(OH) | Chen et al., (1998)  Koca et al. (2007)  Aykas and Rodriguez-Saona (2016) | 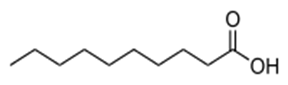 |
| Lauric acid (C12) | 2921, 2855  1745  1460  1175  940-1030 | δas (CH2)  COO-  δ(CH)  δ(CO)  δ(CH) | Chen et al., (1998)  Koca et al. (2007)  Aykas and Rodriguez-Saona (2016) | 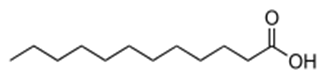 |
| Myristic acid (C14) | 2921, 2855  1745  1460  1175  940-1030 | δas (CH2)  COO-  δ(CH)  δ(CO)  δ(CH) | Chen et al., (1998)  Koca et al. (2007)  Aykas and Rodriguez-Saona (2016) | 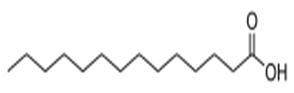 |
| Palmitic acid (C16) | 1745  1460  1175  940-1030 | δas (CH2)  COO-  δ(CH)  δ(CO)  δ(CH) | Chen et al., (1998)  Koca et al. (2007)  Aykas and Rodriguez-Saona (2016) | 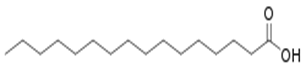 |
| Stearic acid (C18) | 2921, 2855  1745  1460  1175  1030-1170 | δas (CH2)  COO^-^  δ(CH)  δ(CO)  δ(CH) | Chen et al., (1998)  Koca et al. (2007)  Aykas and Rodriguez-Saona (2016) | 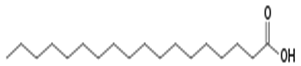 |
| Oleic acid (C18:1) | 2921, 2855  1700-1750  1460  1175  1040-1120 | δas (CH2)  COO-, δ(C=O)  δ(CH)  δ(CO)  δ(CH) | Chen et al., (1998)  Koca et al. (2007)  Aykas and Rodriguez-Saona (2016) | 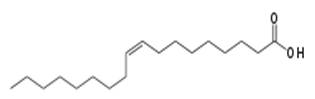 |
| Linoleic acid (C18:2) | 2921, 2855  1700-1750  1460  1175  940-1030 | δas (CH2)  COO-, δ(C=O)  δ(CH)  δ(CO)  δ(CH), δ(OH) | Chen et al., (1998)  Koca et al. (2007)  Aykas and Rodriguez-Saona (2016) | 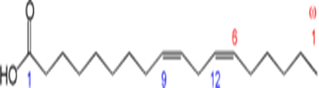 |

**REFERENCES**

Aykas DP, Rodriguez-Saona LE. **Assessing potato chip oil quality using a portable infrared spectrometer combined with pattern recognition analysis. *Anal. Methods*. (**2016) **8:**731-741. https://doi.org/10.1039/C5AY02387D

Barth A. The infrared absorption of amino acid side chains. *Prog Biophys Mol Biol*. (2000) 74(3-5):141-73. doi: 10.1016/s0079-6107(00)00021-3. PMID: 11226511.

Berendzen J, Braunstein D. Temperature-derivative spectroscopy: A tool for protein dynamics. *Proc. Natl. Acad*. *Sci.* USA. (1990). 87:1–5.

Caswell DS, Spiro TG. Proline signals in ultraviolett resonance Raman spectra of proteins: Cis-trans isomerism in polyproline and ribonuclease A. *J. Am. Chem. Soc*. (1987). 109:2796–2800. https://doi.org/10.1021/ja00243a037

Chen M, Irudayaraj J, McMahon DJ. Examination of full fat and reduced fat Cheddar cheese during ripening by Fourier transform infrared spectroscopy. *J. Dairy Sci.* (1998). 81:2791-2797. https://doi.org/10.3168/jds.S0022-0302(98)75837-0

Chirgadze YN, Fedorov OV, Trushina NP. Estimation of amino acid residue side chain absorption in the infrared spectra of protein solutions in heavy water. *Biopolymers.* (1975). 14:679–694. https://doi.org/10.3168/jds.S0022-0302(98)75837-0

Colthup NB, Daly LH, Wiberley SE. Introduction to Infrared and Raman Spectroscopy, 2nd Edition. *Academic Press*, New York. 1975

Dhamelincourt P, Ramirez FJ. Polarized micro-Raman and FT-IR spectra of l-glutamine. *Appl. Spectrosc*. (1993). 47:446–451.

Dollinger G, Eisenstein L, Shuo-Liang L, Nakanishi K, Termini J. Fourier transform infrared diﬀerence spectroscopy of bacteriorhodopsin and its photoproducts regenerated with deuterated tyrosine. *Biochemistry* (1986). 25:6524–6533. DOI: 10.1021/bi00369a028

Fabian H, Yuan T, Vogel HJ, Mantsch HH. Comparative analysis of the amino- and carboxy-terminal domains of calmodulin by Fourier transform infrared spectroscopy. *Eur. Biophys. J.* (1996). 24:195–201. Doi: 10.1007/bf00205100

Gerwert K, Hess B, Engelhard M. Proline residues undergo structural changes during proton pumping in bacteriorhodopsin. *FEBS Lett*. (1990) 261:449–454. https://doi.org/10.1016/0014-5793(90)80613-N

Hasegawa K, Ono TA, Noguchi T. Vibrational spectra and ab initio DFT calculations of 4-methylimidazole and its diﬀerent protonation forms: infrared and Raman markers of the protonation state of a histidine side chain. *J. Phys. Chem. B.* (2000). 104:4253–4265. https://doi.org/10.1021/jp000157d

Hienerwadel R, Boussac A, Breton J, Diner B, Berthomieu C. Fourier transform infrared diﬀerence spectroscopy of photosystem II tyrosine D using site-directed mutagenesis and speciﬁc isotope labelling. *Biochemistry*. (1997). 36(14):712–14,723. DOI: 10.1021/bi971521a

Lagant P, Vergoten G, Peticolas WL. On the use of ultraviolett resonance Raman intensities to elaborate molecular force ﬁelds: application to nucleic acid bases and aromatic amino acid residues models. *Biospectroscopy.* (1998). 4:379–393. https://doi.org/10.1002/(SICI)1520-6343(1998)4:6<379::AID-BSPY3>3.0.CO;2-2

Lautie´ A, Lautie´ MF, Gruger A, Fakhri SA. Etude par spectrome´ trie i.r. et Raman de l’indole et de l’indolizine. Liaison hydroge` ne NH *Spectrochim. Acta A*. (1980). 36:85–94. Doi: 10.1016/0584-8539(80)80062-6

Lewis RNAH, McElhaney RN. Fourier transform infrared spectroscopy in the study of hydrated lipids and lipid bilayer membranes. In: Mantsch, H.H., Chapman, D. (Eds.), *Infrared Spectroscopy of Biomolecules*. Wiley, New York, (1996). p. 159–202.

Madec C, Lauransan J, Garrigou-Lagrange C. Etude du spectre de vibration de la dl-serine et des ses de´ rive´ s deuteries. *Can. J. Spectrosc*. (1978). 23:166–172. DOI:10.1051/JCP/1970670757

Noguchi T, Fukami Y, Oh-oka H, Inoue Y. Fourier transform infrared study on the primary donor P798 of Heliobacterium modesticaldum: cysteine S-H coupled to P798 and molecular interactions of carbonyl groups. *Biochemistry*. (1997). 36:12329–12336. DOI: 10.1021/bi970853c

Overman SA, Thomas GJ. Raman markers of nonaromatic side chains in an a-helix assembly: Ala, Asp, Glu, Gly, Ile, Leu, Lys, Ser, and Val residues of phage fd subunits. *Biochemistry*. (1999). 38:4018–4027. https://doi.org/10.1021/bi982901e

Paucean A, Vodnar DC, Muresan V, Fetea F, Ranga F, Man S, Muste S, Socaciu C. Monitoring lactic acid concentrations by infrared spectroscopy: A new developed method for Lactobacillus fermenting media with potential food applications. *Acta Alimentaria*. (2017). 46:420-427. 10.1556/066.2017.0003.

Petković M, Novak J, Došlić N. Shaping the infrared spectrum of the acetic acid dimer in the OH-stretching range: Multiple conformers and anharmonic coupling. *Chemical Physics Letters,* (2009). 474(4-6):248-252. https://doi.org/10.1016/j.cplett.2009.04.014.

Pinchas S, Laulicht I. Infrared Spectra of Labelled Compounds. Academic Press, London. 1971.

Rahmelow K, Hu¨ bner W, Ackermann T. Infrared absorbances of protein side chains. *Anal. Biochem.* (1998). 257:1–11. DOI: 10.1006/abio.1997.2502

Ramachandran E, Natarajan S. Gel growth and characterization of β-DL-methionine. *Cryst.* *Res. Technol*. (2006) 41(4):411 – 415 / DOI 10.1002/crat.200510595

Rothschild KJ, He YW, Gray D, Roepe PD, Pelletier SL, Brown RS, Herzfeld J. Fourier transform infrared evidence for proline structural changes during the bacteriorhodopsin photocycle. *Proc. Natl. Acad. Sci.* USA. (1989). 86:9832–9835.

Sengupta PK, Krimm S. Vibrational analysis of peptides, polypeptides, and proteins. XXXII. a-poly(l- glutamic acid). *Biopolymers.* (1985). 24: 1479–1491. https://doi.org/10.1002/bip.360240805

Susi H, Byler DM, Gerasimowicz WV. Vibrational analysis of amino acids: cysteine, serine, b-chloroalanine. *J. Mol. Struct*. (1983). 102: 63–79. https://doi.org/10.1016/0022-2860(83)80007-6

Takeuchi H, Harada I. Normal coordinate analysis of the indole ring. *Spectrochim. Acta* *A*. (1986). 42:1069–1078. https://doi.org/10.1016/0584-8539(86)80021-6

Takeuchi H, Watanabe N, Harada I. Vibrational spectra and normal coordinate analysis of p-cresol and its deuterated analogs. *Spectrochim. Acta A*. (1988). 44:749–761.

Tarhan I, Ashraf A, Kara IH. Quantitative determination of free fatty acids in extra virgin olive oils by multivariate methods and Fourier transform infrared spectroscopy considering different absorption modes. *Int. J Food Properties*. (2017). 20(1):790-797. DOI: 10.1080/10942912.2017.1312437

Umadevi M, Thomas AE. Fourier transformed infrared spectral investigations of molecular interactions in propionic acid–2-propanol binary system. *Spectrochimica Acta Part A: Molecular and Biomolecular Spectroscopy.* (2010) 75(4): 1181-1190. https://doi.org/10.1016/j.saa.2009.08.022.

Venyaminov SY, Kalnin NN. Quantitative IR spectrophotometry of peptide compounds in water (H2O) solutions. I. Spectral parameters of amino acid residue absorption bands. *Biopolymers*. (1990). 30:1243–1257. DOI: 10.1002/bip.360301309

Wright W, Vanderkooi JM. Use of IR absorption of the carboxyl group of amino acids and their metabolites to determine pKs, to study proteins, and to monitor enzymatic activity. *Biospectroscopy*. (1997). 3:457–467. https://doi.org/10.1002/(SICI)1520-6343(1997)3:6<457::AID-BSPY5>3.0.CO;2-YCitations: 17
